# The Opening Events of the New Session

**Published:** 1920-10-16

**Authors:** 


### INCORPORATED ASSOCIATION OF HOSPITAL OFFICERS.

### The Opening Events of the New Session.

On Friday, October 8, Mr. George Watts, the new President of the Hospital Officers' Association, delivered the Presidential address at the Headquarters at 28 Bedford Square, W.C. Mr. George Watts is one of the oldest members of the Association, and has worked strenuously in its behalf during many years, having served as Editor of the *Hospital Gazette*, Chairman of the Executive Council, and in other capacities. There are few hospital officers who know more about hospitals than Mr. Watts, and especially valuable is anything that comes from him as representing the point of view of the hospital worker, which is too often left unexpressed when those interested in hospital matters meet together under existing organisations for the discussion of topical matters. King Edward's Hospital Fund for London is an entirely autocratic and independent body, no doubt rightly so when simply the distribution of money is concerned. The British Hospitals Association may be fairly described as a large debating society, in which, if only by courtesy, Chairmen, Committee-men, honorary medical officers and others not constantly and directly concerned in every-day administrative duties, take first place in the proceedings. It is, moreover, a body which, when undertaking certain collective executive duties (for there is no other Society to which such functions can conveniently be relegated), too obviously betrays the fact that it suffers greatly from the lack of an inside view of hospital conditions and needs. When, again, a group of hospitals get together and take a line of action, as was recently the case in respect of the wages of unskilled workers, they, chiefly because they are not representative of the whole hospital world, err in a particularly exasperating way by tying the hands of others who have had no voice in the matter.

### A Proposed New Central Board.

Such were the arguments of Mr. Watts in his Presidential Address, and on them he made a plea for a Central Board composed of Committee-men and actual workers. This Board, he suggested, should be elected by the British Hospitals Association, as regards voluntary workers, and by the Hospital Officers' Association in respect of salaried administrations, in both cases by the regional scheme such as that now being organised by the former Association. Such a body, Mr. Watts held, would be in a peculiarly favourable position to advise hospitals as to joint action in matters of general concern, having first-hand information of a valuable nature that it would be impossible to gather quickly and efficiently by the old methods. The speakers who joined in the debate which followed the President's able paper, some of whom hailed from provincial industrial centres, warmly agreed with the main lines of his thesis, and expressed the hope that steps would immediately be taken to ascertain the general opinion of the hospital world in its respect. No doubt many hospital workers will be keenly interested in a scheme which is so much in accordance with the modern feeling that it is all to the good of an industry, or any other field of endeavour, to make use of the practical opinions of actual workers to perfect the organisation. On the day following the address a visit was made by the members of the Hospital Officers' Association to the City of London Hospital for Diseases of the Chest, Victoria Park, where Mr. George Watts is Secretary, and after an instructive inspection the annual service was held in the chapel, where a helpful address was given by the Rector of Bow. The preacher dealt largely with a plea that hospital employees were particularly singled out as those who could in their conduct and their work appear as witnesses to the example of the Founder of the Christian Church.

Very little is known of Mr. F. W. Ordish, the original architect of the City of London Hospital for Diseases of the Chest, Victoria Park. The very beautiful building he erected in 1870 has been enlarged, but otherwise externally unchanged. His work inside was prior to present-day constructional ideas, especially in respect of the treatment of tuberculosis; therefore a great deal of the ornamentation—Moresque ceilings, elaborate capitals, and other features—unfavourably impress the visitor until he realises that they are chiefly in parts of the buildings not used by the patients. The new portions, mostly from the plans of Mr. W. A. Pite, are as perfect as skill can make them; the old parts have been adapted in a skilful way, notably by the removal of screens, adding, amongst other effects, increased airiness and space to the day-rooms. Throughout the building there is a uniform effect of air, light, and spaciousness, which is thoroughly in keeping with its purpose, and on a bright, clear day, such as when the members of the Hospital Officers' Association made their visit, this condition is particularly noticeable and agreeable. There is still to be seen on each floor the general drinking-fountain provided by a former unenlightened management for the refreshment of the patients; these have for many years been covered in and remain merely as relics of the Dark Ages. Beyond the spacious grounds of the hospital stretches the extensive area so dear to the East End—Victoria Park—a splendid help to the physicians of this excellent institution, and a feature which one notices is wisely never left unmentioned in the reports and appeals.

### The Education of Hospital Workers.

Amongst forthcoming events in the sessional programme of the Hospital Officers' Association we notice some important features. On December 10 Viscount Hambleden will address the members on the question of contributions by patients, and the experience of King's College Hospital will no doubt be made available to many who are now making experiments in this direction. We understand that the present yield from this source at King's College Hospital is £70 a month. On November 12 the views of Mr. Morris, the House Governor of the London Hospital, on the subject of consultant and other medical services, and on the report of the Consultative Committee of the Ministry of Health, will be placed before the members of the Association. The educational section of the Association's work will probably include an extension to the almoner's or social side of hospital work. At present the most excellent school at St. Thomas's Hospital is the only institution for methodical training of almoners. The movement in the direction of patients' payments has emphasised the need for an almoner, inquiry officer, or social worker in each hospital, and the demand is now several years ahead of the supply, with the effect that an entirely home-made training is often the only one available. Moreover, the Almoners' Council's scheme, although most efficient as regards training, excludes, by reason of its expensiveness, many suitable women already engaged in hospital work whose practical experience and suitable personality are to some extent wasted. No doubt the Association will in due time publish particulars of the new scheme of training, which will, we are sure, be eagerly studied by both employers and employed.

